# Designer Amphiphilic Short Peptides Enhance Thermal Stability of Isolated Photosystem-I

**DOI:** 10.1371/journal.pone.0010233

**Published:** 2010-04-21

**Authors:** Baosheng Ge, Feng Yang, Daoyong Yu, Shuang Liu, Hai Xu

**Affiliations:** Center for Bioengineering and Biotechnology, China University of Petroleum, Qingdao, People's Republic of China; Massachusetts Institute of Technology, United States of America

## Abstract

Stability of membrane protein is crucial during protein purification and crystallization as well as in the fabrication of protein-based devices. Several recent studies have examined how various surfactants can stabilize membrane proteins out of their native membrane environment. However, there is still no single surfactant that can be universally employed for all membrane proteins. Because of the lack of knowledge on the interaction between surfactants and membrane proteins, the choice of a surfactant for a specific membrane protein remains purely empirical. Here we report that a group of short amphiphilic peptides improve the thermal stability of the multi-domain protein complex photosystem-I (PS-I) in aqueous solution and that the peptide surfactants have obvious advantages over other commonly used alkyl chain based surfactants. Of all the short peptides studied, Ac-I_5_K_2_-CONH_2_ (I_5_K_2_) showed the best stabilizing effect by enhancing the melting temperature of PS-I from 48.0°C to 53.0°C at concentration of 0.65 mM and extending the half life of isolated PS-I significantly. AFM experiments showed that PS-I/I_5_K_2_/Triton X-100 formed large and stable vesicles and thus provide interfacial environment mimicking that of native membranes, which may partly explain why I_5_K_2_ enhanced the thermal stability of PS-I. Hydrophobic and hydrophilic group length of I_x_K_y_ had an important influence on the stabilization of PS-I. Our results showed that longer hydrophobic group was more effective in stabilizing PS-I. These simple short peptides therefore exhibit significant potential for applications in membrane protein studies.

## Introduction

Membrane proteins offer exciting opportunities for the development of biomimetic solar energy harvesting devices and can be used for fabricating sensitive biosensors for the detection of odors, diseases and chemicals. However, the instability of membrane proteins has significantly impeded fundamental research on this class of proteins and largely limited their application [1]. In order to base devices on these proteins, it is crucial to obtain stable, functional membrane proteins. Extensive studies have revealed that after being taken out of their native membrane environment membrane proteins are not stable and are extremely prone to structural deformation and loss of activity [2]. Some new surfactants show promise in the stabilization of membrane proteins [3] but there are no general rules for the selection of surfactants for an individual membrane protein. This selection remains purely empirical because we are far from fully understanding the interaction between surfactants and membrane proteins [4], [5], [6]. Most surfactants so far used can only extend the stability of membrane proteins to a limited extent and are not feasible for practical application.

Photosystem-I (PS-I) is a membrane protein complex that is associated with electron transport and exists as a large, multi-subunit complex with dozens of transmembrane spanning domains [7]. Light harvesting and photoelectron conversion efficiency of PS-I can exceed 95% during photosynthesis in green plants or algae, making it attractive for the development of a PS-I based biophotovoltaic device for solar energy conversion [8]. Both PS-I and PS-II have been isolated and integrated into solid state electrodes to develop photosystem based biophotovoltaic devices, and reasonable currents have been detected [9], [10]. Although Das et al [9] have demonstrated that surfactants can improve PS-I stability, the optimal conditions for improving PS-I stability need to be further explored. Work by Matsumoto et al [1] and Kiley et al [2] have demonstrated that under similar conditions peptide surfactants offer better stabilization than commonly used alkyl chain surfactants, making it worthwhile to examine the effects of molecular architecture of peptide surfactants on membrane protein stabilization.

Yeh et al [11] and Zhao et al [12] have examined the stabilization effect of amphiphilic peptides on glycerol-3-phosphate dehydrogenase and bovine rhodopsin. The results show that the amphiphilic peptide A_6_K (Ac-A_6_K-CONH_2_) can enhance the stability and functionality of glycerol-3-phosphate dehydrogenase, and that A_6_D (Ac-A_6_D-COOH) can enhance the thermal stability of bovine rhodopsin and significantly extend its half life, even under thermal denaturation conditions. They have shown that these short peptides are much better than commonly used alkyl chain surfactants in stabilizing membrane proteins and show promise as stabilizing agents in membrane protein based devices.

Photosynthesis activity of green plants is significantly inhibited when the environmental temperature is above 40°C, probably due to dimerization of red form chlorophylls and decrease of the efficiency of electron transfer from antenna to reaction center [13], [14], [15]. For a solar energy conversion device, the working temperature is often above ambient when exposed to sunlight in summer. It is therefore essential to improve the thermal stability of PS-I so that it can be used in photosynthetic solar cell development.

Here we report a study on the effect of a group of amphiphilic short peptides (7-8 residues) on the thermostability of the PS-I membrane protein complex extracted from *Spirulina platensis*. The thermostability of PS-I has been characterized with circular dichroism. Since I_5_K_2_ was found to enhance the thermostability of PS-I, we have systematically examined the effect of the number of lysine and isoleucine in I_x_K_y_ to try to optimize the peptide design.

## Results

### Thermal stability

**Figure 1:**
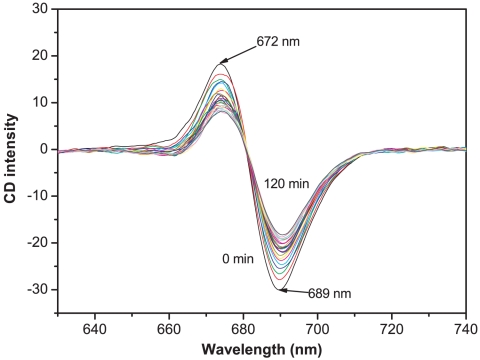
Time dependence of the CD spectrum of PS-I at 48°C. The sample ([Chla] = 22 µM) was incubated at 48°C for 5 min before the experiments. Then spectra were recorded between 630 nm and 740 nm every 5 min for a total time of 120 min using 10 mm pathlength quartz cuvette.

The CD spectrum of PS-I at 48°C was measured every 5 min. Figure 1 shows two distinct peaks in the CD spectrum within the visible wavelength range, 672–689 nm, arising from red chlorophylls of the PS-I reaction center. This feature has been reported previously [16], [17], [18]. Figure 1 also shows that the CD spectrum changed significantly with time, suggesting that at this temperature the conformation of PS-I must also have changed with time. Also, the signal at the two peaks changed more significantly than the other regions and the largest changes occurred at 689 nm. We therefore used the signal at 689 nm to characterize the stability of PS-I in the presence of amphiphilic peptides.

**Figure 2:**
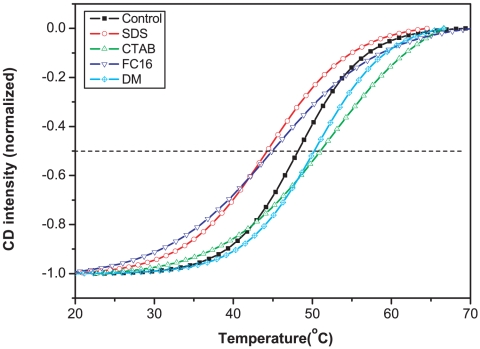
Thermal stability of PS-I measured by following CD intensity at 689 nm versus temperature in the presence of different surfactants (0.5% (w/v)). The sample with 0.8 mM nonionic Triton X-100 (to solubilize PS-I) acted as a control. All the data were taken after protein solutions were incubated for 5 min. Curves were fitted with equation 1.

Figure 2 shows as control the variation of the 689 nm CD signal in Triton X-100. The mid point of the change corresponds to the melting temperature and a value of 48.0°C was obtained by fitting equation (1). Alkyl chain surfactants have been used in the stabilization of membrane proteins [1], [2], [17], [18], [19] and we selected four representative surfactants and checked their influence on the stability of PS-I at a fixed concentration of 0.5% (w/v). The melting temperature of PS-I decreased from 48.0°C to 44.2°C and 44.8°C on the addition of anionic SDS (sodium dodecyl sulfate) and zwitterionic FC-16 (Fos-choline-16, hexadecylphosphocholine) respectively. However, cationic CTAB (cetyltrimethyl ammonium bromide) and non-ionic DM (n-decyl-β-D-maltopyranoside) improved the stability of PS-I, increasing the melting temperature to 50.6°C and 50.4°C respectively.

**Figure 3:**
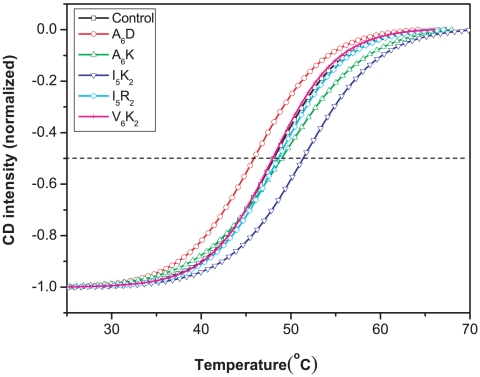
Thermal stability of PS-I in the absence and presence of amphiphilic short peptides. Data determination and analysis were kept the same as in Figure 2. Sigmoidal curves were normalized to the lowest point and fitted with equation 1.

The short peptides A_6_K (Ac-A_6_K-CONH_2_), A_6_D (Ac-A_6_D-COOH), I_5_K_2_, I_5_R_2_ (Ac-I_5_R_2_-CONH_2_) and V_6_K_2_ (Ac-V_6_K_2_-CONH_2_), can also affect the thermal stability of PS-I, as shown in Figure 3. At a concentration of 0.55 mM I_5_K_2_ increased the melting temperature of PS-I from 48.0°C to 51.2°C, whilst the three peptides I_5_R_2_, A_6_K and V_6_K_2_ showed little difference from the control. Interestingly, 0.55 mM of A_6_D decreased the melting temperature of PS-I to 45.8°C in sharp contrast to its significant enhancement of the thermal stability of bovine rhodopsin [12]. This shows that different proteins may be affected differently by the same surfactant and that the mechanism of stabilization is complex.

**Figure 4:**
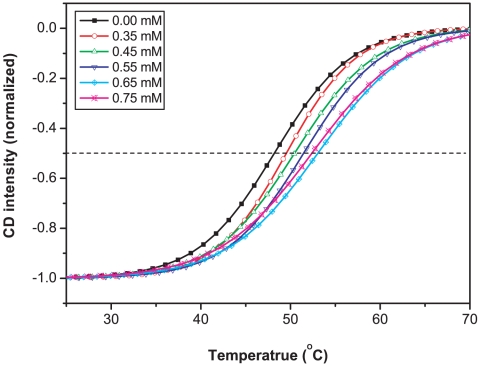
Temperature dependence of CD signal of PS-I at 689 nm as a function of I_5_K_2_ concentration. Sigmoidal curves were normalized to the lowest point. Data determination and analysis were the same as in Figure 2. Curves were fitted with equation 1.

**Figure 5:**
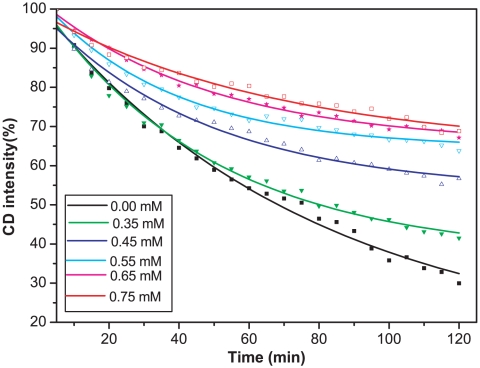
Thermal denaturation kinetics of PS-I at different I_5_K_2_ concentration. Circular dichroism spectra were measured every 5 min for totally 120 min at 48°C with a fixed wavelength of 689 nm. Curves were fitted to equation 2.

The effect of I_5_K_2_ concentration on the thermal stability of PS-I was then examined. Figure 4 shows that increase in I_5_K_2_ concentration led to a steady increase of melting temperature. In the presence of 0.65 mM of I_5_K_2_, the melting temperature increased to 53.0°C. The time-resolved CD experiments shown in Figure 5 further show that the thermal half-life of PS-I increased from 67 min without I_5_K_2_ to 101 min at the I_5_K_2_ concentration of 0.35 mM, and to 173 min at a concentration of 0.45 mM. A higher CD intensity at 689 nm was observed with further increase of I_5_K_2_ concentration but the stabilization effect seemed to weakening. These observations show that the peptide I_5_K_2_ enhances the thermal stability of isolated PS-I and significantly extends its lifetime.

### Circular dichroism and AFM imaging

**Figure 6:**
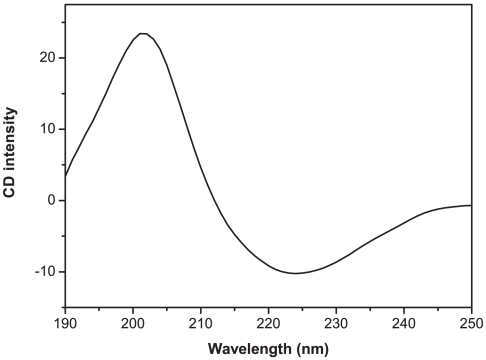
CD spectrum of I_5_K_2_ in MES buffer (pH 6.5). The spectrum was measured at 25.0°C with 1 mm path-length. Concentration of peptide was adjusted to 0.55 mM in MES buffer (pH 6.5). The spectrum was recorded between 190 and 250 nm with 1 nm resolution and a 2 s averaging time. The final spectrum was background-corrected by subtracting the corresponding buffer spectra obtained under identical conditions.

The CD spectrum of I_5_K_2_ in MES buffer at pH 6.5, shown in Figure 6, shows one positive maximum at 203 nm and one negative maximum at 224 nm, indicating the existence of a stable parallel β-sheet structure. It has been reported that Ac-I_6_K_2_-CONH_2_ forms β-sheet structures in solution and flat ribbon-like or sheet-like structures on mica, and that these structures are stable up to 90°C [20].

**Figure 7:**
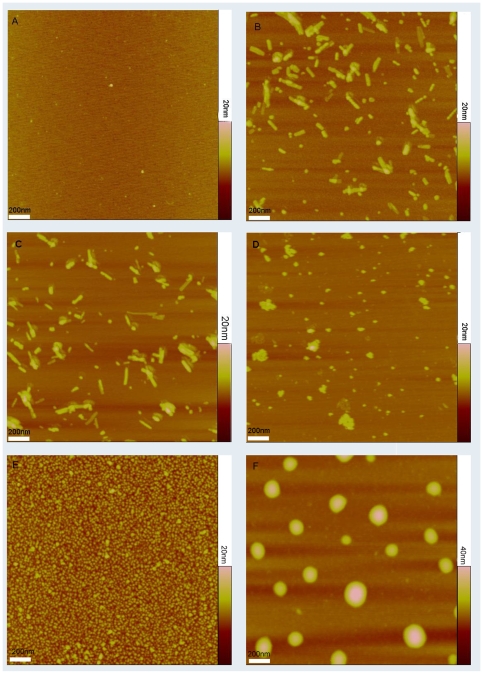
AFM images of Triton X-100, PS-I, I_5_K_2_ peptide and their mixture. A: Triton X-100 on mica surface; B: I_5_K_2_; C: Triton X-100 and I_5_K_2_ mixture on mica surface; D: PS-I along on mica; E: PS-I in the presence of Triton X-100; F: PS-I in the presence of Triton X-100 and I_5_K_2_.

The topological structures of PS-I, with and without peptides, were imaged on the surface of mica using tapping mode AFM. Figure 7A shows that Triton X-100 alone absorbed on mica slightly but did not form any observable aggregates on mica surface under the experimental conditions. I_5_K_2_ in MES buffer at pH 6.5 formed short nanorods (Figure 7B). Mixing Triton X-100 with I_5_K_2_ does not change this situation (Figure 7C). AFM experiments suggest that in the absence of surfactants, PS-I molecules on mica surface were monomers, trimers or larger aggregates with diameters ranging from 15 nm to over 100 nm but a height of about 6 nm (Figure 7D). PS-I monomer and trimer complex have similar estimated heights of 6–9 nm but different diameters of ca. 15 nm and 50 nm respectively [1], [21]. When PS-I was mixed with 0.47 mM Triton X-100 (Figure 7E), PS-I dissolved and assembled mainly into trimers with an average diameter of 40 nm and a height of 7 nm. However, PS-I/I_5_K_2_/Triton X-100 mixtures had features in the form of large vesicles with step heights of 10–18 nm (average 16 nm) and an average diameter of 140 nm with particle sizes ranging between 100 and 220 nm (Figure 7F). The mechanism of formation of these vesicles is not known. As Triton X-100 is highly effective at dispersing PS-I into trimers [22], it probably binds strongly to PS-I and disperses these molecules into MES buffer. The subsequent mixing with I_5_K_2_ might lead to the formation of vesicles that help protect PS-I from denaturation.

### Thermal stability with I_x_K_y_

**Figure 8:**
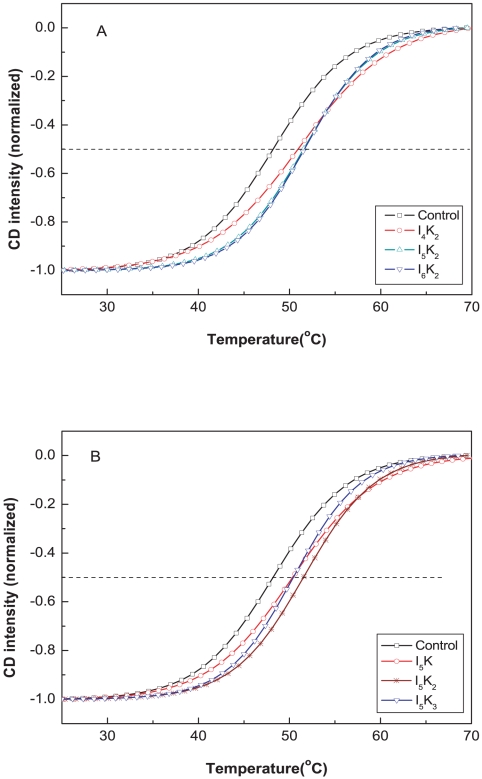
Effect of the number of lysine and isoleucine in I_x_K_y_ on the thermal stability of PS-I. A: thermal stability of PS-I with I_x_K_2_; B: thermal stability of PS-I with I_5_K_y_. Data determination and analysis were same with Figure 2. Sigmoidal curves were normalized to the lowest point and fitted with equation 1.

The amphiphilicity of I_5_K_2_ is strongly influenced by the number of lysine and isoleucine in the head and tail regions. The influence of varying lysine and isoleucine content on the stabilizing capability and the melting temperature of PS-I of six different peptides, Ac-IIIK-CONH_2_ (I_3_K), Ac-IIIIK-CONH_2_ (I_4_K), Ac-IIIIIK-CONH_2_ (I_5_K), Ac-IIIIKK-CONH_2_ (I_4_K_2_), Ac-IIIIIKKK-CONH_2_ (I_5_K_3_), Ac-IIIIIIKK-CONH_2_ (I_6_K_2_) was also measured. In the series of I_3_K, I_4_K and I_5_K, I_5_K showed the strongest stabilizing effect, suggesting that a longer hydrophobic tail is more effective. I_4_K_2_, I_5_K_2_ and I_6_K_2_ showed a similar trend, i.e. I_4_K_2_<I_5_K_2_<I_6_K_2_ with I_6_K_2_ increasing the melting temperature to 51.6°C, as shown in Figure 8A. It is probable that the more hydrophobic peptide surfactants penetrate more extensively into the hydrophobic regions formed by Triton X-100 and PS-I and therefore provide better protection.

For peptides containing different numbers of lysine, i.e. I_5_K, I_5_K_2_ and I_5_K_3_ Figure 8B shows that the melting temperature of PS-I was 50.2°C, 51.4°C and 50.4°C in 0.55 mM of I_5_K, I_5_K_2_ and I_5_K_3_ respectively. This result suggests that longer hydrophilic heads do not increase the stabilizing ability further and that there is an optimal hydrophilic head size.

Δ*H* is another useful parameter. Δ*H* at *T*_m_ for the interaction of PS-I with different alkyl chain surfactants was found to be in the range of 120 to 240 kJ/mol (Table S1). Δ*S* for the same interaction was found to be in the range of 380 to 600 J/mol·K, smaller in general than the value, 510 to 750 J/mol·K for the peptides.

## Discussion

The peptide I_5_K_2_ was found to enhance the thermal stability of PS-I in solution. Five different types of related peptide surfactants were also tested, A_6_D, A_6_K, I_5_K_2_, I_5_R_2_ and V_6_K_2_. Their effect on PS-I stability was to stabilize for PS-I in the order I_5_K_2_>A_6_K>I_5_R_2_>V_6_K_2_> control >A_6_D. The positively charged K series was more effective than negatively charged D series for PS-I. A similar charge dependence on stabilization was observed for alkyl chain surfactants, which has been correlated with the surface charge of PS-I [24]. For different types of hydrophobic tails, the results showed no obvious systematic effect on stabilization of PS-I. Changing the number of lysine or isoleucine changes the amphiphilicity of the peptides, which alters their interactions with water and membrane proteins. Our results show that increasing the number of isoleucines increases the stabilizing effect of I_x_K_2_ on PS-I. However, the longer hydrophobic group reduces the solubility and makes it harder to achieve the desired concentration. Changing the number of lysines changes the interaction balance of PS-I, peptides and water. Too long or too short a hydrophilic group length does not give maximum stabilization, due to the balance between solubility and the capacity for hydrophobic binding. Thus, there are optimal lengths for the hydrophobic tail and hydrophilic head regions for a specific membrane protein.

It has been proposed that surfactants can mimic lipid membrane environment to some extent and hence stabilize membrane proteins and keep their activities *in vitro* [25]. The amphiphilic nature of these short peptides allow formation of large structural architectures through self-assembly, which can encapsulate the PS-I complexes. We have shown that peptide amphiphiles such as I_5_K_2_ can form stable β-sheets and the secondary structure is strong and robust against temperature increase, consistent with observations by Baumann et al [20]. It is probable that these structural features are important for them to participate in the formation of large vesicles and enhance the thermal stability of PS-I. Some short peptides, such as V_6_K_2_ and A_6_D, showed little effect or a negative effect on the stabilization of PS-I. This might arise from the intrinsic structural mismatch in promoting the formation of vesicular structure and the weak interaction between peptide surfactants and the protein [1].

It should be noted that the determination of *T*_m_ values in protein denaturation is based on the assumption that the system is in reversible equilibrium. In the presence of irreversible process, it is hard to determine accurate *T*_m_ values, unless the irreversible process is much slower than the melting process, i.e. the thermal melting experiments can be finished in a much shorter time than the half-life of the protein. When the rate of the irreversible process is comparable with the rate of temperature increase, the apparent *T*_m_ value will be smaller than the real *T*_m_ value. In the present work, the half-life of PS-I at 48°C was in the range of 67 to 173 min, while the temperature was increased at 1°C/min. A negative deviation of apparent *T*_m_ is therefore expected. In principle, it is possible to minimize the deviation by increasing the temperature much faster. However, to obtain homogenous and accurate temperature, the rate of increase of temperature was limited to 1°C/min. The *T*_m_ values reported here were apparent *T*_m_ values and should not be interpreted as a parameter for thermodynamic stability, i.e. to calculate the Δ*G*_D-N_, but it is still useful as a parameter to check the influence of the peptides on the thermal stability of PS-I, which was determined by both thermodynamics and kinetics.

## Materials and Methods

### PS-I purification

The PS-I complex was extracted from the thylakoid membranes of *Spirulina platensis* following the procedures described previously [26]. Algae cells were harvested and suspended in STNMC solution (300 mM sucrose, 10 mM NaC1, 5 mM MgCl_2_, 5 mM CaCl_2_, 50 mM Tris-HCl and pH 7.8) with protease inhibitor phenylmethyl sulfonylfluoride (PMSF, 20 mM). The cells were sonicated and cell debris was removed by centrifugation at 3000 g for 15 min. The photosynthetic membrane protein complex was then isolated by centrifugation at 50000 g for 60 min. The membranes were washed and solubilized in buffer (50 mM Tris-HCl pH 7.8, 5 mM MgCl_2_, 5 mM CaCl_2_) containing nonionic surfactant Triton X-100 at a concentration 22.5× that of Chla (chlorophyll-a). The supernatant was loaded onto a 10–30% (w/v) step sucrose gradient at 4°C and centrifuged for 16 h at 160000 g. The lower green band was collected and stored at −20°C. The purified PS-I was characterized with SDS-PAGE, fluorescence spectrum and circular dichroism (CD). The chlorophyll content of PS-I was measured as described in [27], [28].

### Chemicals and Peptides

Amphiphilic short peptides, Acetyl-AAAAAAK-CONH_2_ (Ac-A_6_K-CONH_2_, or A_6_K), Acetyl-AAAAAAD-COOH (Ac-A_6_D-COOH, or A_6_D), Acetyl-IIIIIKK-CONH_2_ (Ac-I_5_K_2_-CONH_2_, or I_5_K_2_), Acetyl-VVVVVVKK-CONH_2_ (Ac-V_6_K_2_-CONH_2_, or V_6_K_2_), Acetyl-IIIIIRR-CONH_2_ (Ac-I_5_R_2_-CONH_2_, or I_5_R_2_), Ac-IIIK-CONH_2_ (Ac-I_3_K-CONH_2_, or I_3_K), Ac-IIIIK-CONH_2_ (Ac-I_4_K-CONH_2_, or I_4_K), Ac-IIIIIK-CONH_2_ (Ac-I_5_K-CONH_2_, or I_5_K), Ac-IIIIKK-CONH_2_ (Ac-I_4_K_2_-CONH_2_, or I_4_K_2_), Ac-IIIIIKKK-CONH_2_ (Ac-I_5_K_3_-CONH_2_, or I_5_K_3_), Ac-IIIIIIKK-CONH_2_ (Ac-I_6_K_2_-CONH_2_, or I_6_K_2_) were synthesized on CEM Liberty Microwave Peptide Synthesizer (Matthews, USA) using the solid phase Fmoc process and purified by HPLC. DM and SDS were purchased from Sigma Aldrich. FC-16 was purchased from Anatrace (USA), and CTAB was obtained from BBI (China). Other chemical reagents were all of analytical grade.

### Thermal stability of PS-I

The absorption of red form chlorophylls is strongly dichroic and the conformational changes can be detected with visible CD signal between 600–760 nm [17]. The decrease of CD signal in this region indicates the dimerization of red form chlorophylls and a decrease in the efficiency of electron transfer from antenna to reaction center [14], [15]. This CD signal is more specific and sensitive than that at 190–250 nm. The thermal stability of the PS-I protein complex was studied on a Bio-Logic MOS-450 CD spectrometer. The temperature was automatically controlled between 25°C and 70°C through a Peltier device. PS-I was suspended in 20 mM MES buffer containing 0.8 mM Triton X-100 at pH 6.5 to achieve a final Chla of 22 µM. Samples were incubated for 5 min prior to recording CD spectra using 10 mm path-length fused quartz cuvettes. The spectra were recorded in 1 nm steps with an integration time of 0.2 s and a band-pass of 2 nm.

Melting temperatures of PS-I with surfactants or peptides were determined by measuring the CD signal at 689 nm as a function of temperature on a Bio-Logic MOS-450 CD spectrometer [18]. The temperature was increased at rate of 1°C/min from 25°C to 70°C.

The thermal stability of PS-I with different concentrations of peptides was studied by measuring the kinetics of its denaturation. The final peptide concentration was adjusted to 0.35, 0.45, 0.55, 0.65, 0.75 mM, respectively. CD spectra were measured every 5 min for a total of 120 min at 48°C with a fixed wavelength of 689 nm.

For all the above experiments, PS-I solubilized with 0.8 mM Triton X-100 was used as a control.

### Secondary structure determination of peptide

To test if the peptide could form any secondary structure in solution, CD spectra were measured on a Bio-Logic MOS-450 CD spectrometer using a 1 mm path-length cell, equilibrated at 25°C. Concentration of peptide was adjusted to 0.55 mM in MES buffer (pH 6.5). Spectra were recorded between 190 and 250 nm with 1 nm resolution and an average time of 2 s. The final spectra were corrected for background by subtracting the corresponding buffer spectra obtained under identical conditions [16].

### Atomic force microscopy (AFM) imaging

AFM studies of peptide surfactants were performed on Nanoscope IVa (Vecco/Digital Instruments, Santa Barbara, USA) operating in tapping mode [29]. Soft silicon probes were chosen (FESP, Veeco Probes) with a tip radius of <10 nm, mounted on a single-beam cantilever. AFM images of a 2 µL sample deposited onto freshly cleaved mica surface were recorded in air at room temperature. Each sample was allowed to interact with the mica surface for 30 s and then was rinsed with Milli-Q water. Samples on the mica surface were then dried in air and images were acquired immediately. Cantilever deflections were recorded with a cantilever frequency of 250 Hz, horizontal scan rate of 1.2 Hz and 512 samples per line. The images were analyzed by Nanoscope software (Version 5.12r3).

### Data analysis

The *[θ]*_689 nm_ at different time points was presented as the percentage of the original CD value at the start of the experiment and fitted with equation 1 [30], where A_n_ is CD intensity of native state, B_n_ is slope of the native state baseline, A_d_ is CD intensity of denatured state, B_d_ is slope of the denatured state baseline, *C*_p_ is the change of the heat capacity, Δ*H* is the change of enthalpy at *T*_m_.



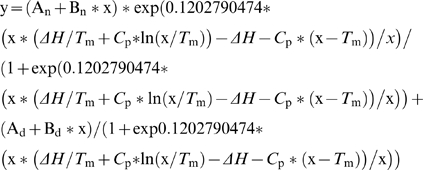
(1)


The half-life of PS-I in each solution was obtained by fitting the relative CD intensity to a single exponential equation with three parameters (Equation 2), and then calculated as equation 3 [12].



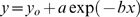
(2)




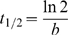
(3)


## Supporting Information

Table S1 *T*_m_, Δ*H* and Δ*S* at *T*_m_ analysis of PS-I with different surfactants.
(0.05 MB DOC)
