# Supplementary material for: Designer Amphiphilic Short Peptides Enhance Thermal Stability of Isolated Photosystem-I
Source: PLoS One. 2010 Apr 21;5(4):e10233. doi: 10.1371/journal.pone.0010233 (PMC2858086; doi:10.1371/journal.pone.0010233)
Supplement: Table S1 — T m, ΔH and ΔS at T m analysis of PS-I with different surfactants. (0.05 MB DOC) [file pone.0010233.s001.doc]

**Supporting information**:

Table S1 *T*_m_, Δ*H* and Δ*S* at *T*_m_ analysis of PS-I with different surfactants

|  | *T*_m_ (K) ^a^ | Δ*H* (kJ/mol) *^b^* | Δ*S*(J/mol·K) ^c^ | Δ*C*_p_ ^d^ |
| --- | --- | --- | --- | --- |
| Control | 321.2 | 207 | 646 | 1275 |
| SDS | 317.4 | 159 | 503 | 987 |
| CTAB | 323.8 | 131 | 406 | 782 |
| FC-16 | 318.0 | 121 | 383 | 751 |
| DM | 323.5 | 192 | 596 | 1180 |
| A_6_D | 318.9 | 215 | 676 | 1350 |
| A_6_K | 322.1 | 184 | 573 | 1125 |
| I_5_K_2_ | 324.4 | 210 | 649 | 1278 |
| I_5_R_2_ | 321.6 | 231 | 721 | 1430 |
| V_6_K_2_ | 320.2 | 240 | 751 | 11719 |
| I_4_K_2_ | 324.1 | 174 | 538 | 1068 |
| I_5_K_2_ | 324.6 | 224 | 690 | 1370 |
| I_6_K_2_ | 324.7 | 232 | 714 | 1416 |
| I_5_K | 323.3 | 189 | 584 | 1148 |
| I_5_K_2_ | 324.6 | 224 | 690 | 1370 |
| I_5_K_3_ | 323.5 | 236 | 732 | 1454 |
| I_5_K_2_(0.35 mM) | 322.6 | 222 | 688 | 1368 |
| I_5_K_2_(0.45 mM) | 323.5 | 194 | 601 | 1194 |
| I_5_K_2_(0.55 mM) | 324.5 | 202 | 624 | 1240 |
| I_5_K_2_(0.65 mM) | 326.1 | 174 | 533 | 1046 |
| I_5_K_2_(0.75 mM) | 325.5 | 167 | 514 | 1061 |

Note: a, *T*_m_ means melting temperature; b, Δ*H* means the change of enthalpy at *T*_m_; c, Δ*S* means the change of entropy at *T*_m_; d, *C*_p_ means the change of the heat capacity. *T*_m_, Δ*H* and *C*_p_ could be obtained by fitting each curve with equation 1, and Δ*S* could be calculated with equation as: Δ*S=*Δ*H/T*_m_.
